# Antisolvent Additive Engineering for Boosting Performance and Stability of Graded Heterojunction Perovskite Solar Cells Using Amide-Functionalized Graphene Quantum Dots

**DOI:** 10.1021/acsami.2c12944

**Published:** 2022-11-29

**Authors:** Elahe Khorshidi, Behzad Rezaei, Arash Kavousighahfarokhi, Jonas Hanisch, Manuel A. Reus, Peter Müller-Buschbaum, Tayebeh Ameri

**Affiliations:** † Department of Chemistry and Center for NanoScience (CeNS), Ludwig-Maximilians-Universität München, Butenandtstrasse 5-13 (E), Munich81377, Germany; ‡ Department of Chemistry, Isfahan University of Technology, Isfahan84156-83111, Iran; § Department of Electrical and Electronic Engineering, Faculty of Engineering, Universiti Putra Malaysia, UPM, Serdang43400, Selangor Darul Ehsan, Malaysia; ∥ Zentrum für Sonnenenergie- und Wasserstoff-Forschung Baden-Württemberg (ZSW), Meitnerstraße 1, Stuttgart70563, Germany; ⊥ Lehrstuhl für Funktionelle Materialien, Physik-Department, Technische Universität München, James-Franck-Straße 1, Garching85748, Germany; # Heinz Maier-Leibnitz Zentrum (MLZ), Technische Universität München, Lichtenbergstr. 1, Garching85748, Germany; ¶ Institute for Materials and Processes, School of Engineering, University of Edinburgh, Sanderson Building, Robert Stevenson Road, EdinburghEH9 3FB, U.K.

**Keywords:** antisolvent additive engineering, graded heterojunction structure, perovskite solar cell, defect passivation, graphene quantum dots

## Abstract

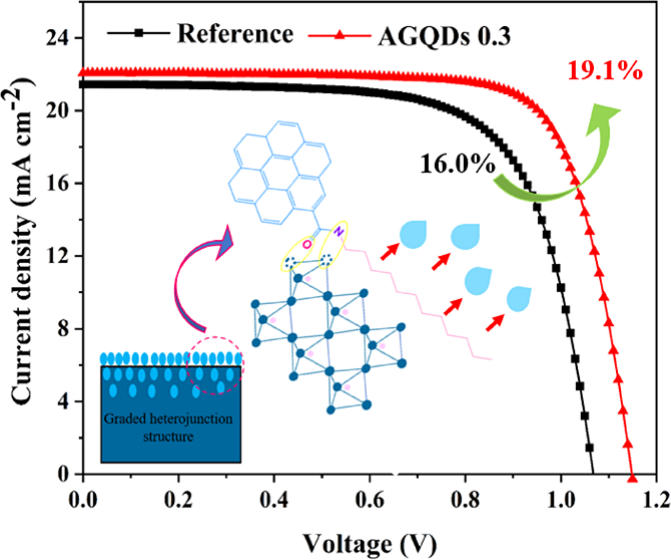

Additive and antisolvent engineering strategies are outstandingly efficient in enhancing perovskite quality, photovoltaic performance, and stability of perovskite solar cells (PSCs). In this work, an effective approach is applied by coupling the antisolvent mixture and multi-functional additive procedures, which is recognized as antisolvent additive engineering (AAE). The graphene quantum dots functionalized with amide (AGQDs), which consists of carbonyl, amine, and long hydrophobic alkyl chain functional groups, are added to the antisolvent mixture of toluene (T) and hexane (H) as an efficient additive to form the CH_3_NH_3_PbI_3_ (MAPI):AGQDs graded heterojunction structure. A broad range of analytical techniques, including scanning electron microscopy, X-ray diffraction, X-ray photoelectron spectroscopy, space charge limited current, UV–visible spectroscopy, external quantum efficiency, and time-of-flight secondary ion mass spectrometry, are used to investigate the effect of AAE treatment with AGQDs on the quality of perovskite film and performance of the PSCs. Importantly, not only a uniform and dense perovskite film with hydrophobic property is obtained but also defects on the perovskite surface are significantly passivated by the interaction between AGQDs and uncoordinated Pb^2+^. As a result, an enhanced power conversion efficiency (PCE) of 19.10% is achieved for the champion PSCs treated with AGQD additive, compared to the PCE of 16.00% for untreated reference PSCs. In addition, the high-efficiency PSCs based on AGQDs show high stability and maintain 89% of their initial PCE after 960 h in ambient conditions.

## 1 Introduction

Since 2009, hybrid perovskite materials have been designed and investigated as outstanding semiconductors for next-generation solar cells due to their unique characteristics, such as easy synthesis, color tunability, high performance, and low-cost processing. Perovskite photovoltaic technology was developed with rapid progress and has achieved a power conversion efficiency (PCE) of over 25% to date.^1−7^ Perovskite materials with the general formula of ABX_3_ (A and B: cation and X: anion) have extraordinary photovoltaic properties, such as high charge carrier mobility, weak exciton binding energy, a high absorption coefficient, and a long charge carrier diffusion length.^8−14^ One of the most important factors in achieving highly efficient perovskite solar cells (PSCs) is the high crystalline quality of perovskite film with minimal grain boundaries and density of trap states, high uniformity, and full coverage.^15−18^

However, polycrystalline perovskite films are deposited based on the solution processing methods, which unavoidably produce a lot of defects at the surface and grain boundaries, such as uncoordinated Pb^2+^ and halide ion (I^–^) vacancy. These grain boundaries and defects not only act as non-radiative recombination centers but also permit moisture and oxygen to diffuse into the perovskite film and reduce the performance and long-term stability of PSCs.^19−22^ Numerous studies have reported an improvement of crystallinity and morphology of perovskite film as well as the passivation of surface and grain boundaries’ defects via various strategies, such as component stoichiometry adjustment, interface engineering, additive implementation, antisolvent engineering, and more.^23−29^ Among these, both additive implementation and antisolvent engineering have turned out to be efficient strategies.^30−34^

Most additive molecules are only able to either improve crystallization or passivate defects.^35,36^ However, bifunctional additives, which contain two functional groups, for example, OH and NH_3_^+^, or OH and Cl^–^, or NH_3_^+^ and COOH, have been demonstrated to enhance the morphology of perovskite film and passivate defects at the surface and grain boundaries, simultaneously.^37−40^ In addition, for defect passivation and improving crystallization, the hydrophobicity of the additive is also essential to boost the air stability of the perovskite film during the fabrication and characterization. Therefore, the development of additives containing not only functional groups as defect passivation agents but also hydrophobic groups is required to improve the efficiency and stability of PSCs, simultaneously.

Although adding additives in perovskite precursors could offer larger grains and fewer defects, the high concentration of additives implemented into perovskite films can lead to a negative effect on the electron and hole transport.^41^ On the other hand, bulk heterojunction structures offer random distribution of additives in perovskite films, which may lead to undesirable contact between electron/hole transport materials and perovskite film.^42−46^ Moreover, since the defects of perovskite film are mostly located at the top surface, most efforts are needed to control defect passivation on the surface.

Recently, adding a functional additive into a perovskite antisolvent, identified as antisolvent additive engineering (AAE), has emerged as another promising approach to boost PCE and prolong the durability of PSCs by forming a graded heterojunction structure.^42,43,47−51^ However, there are only few studies on the AAE approach to date, and a quite limited number of additives have been applied in perovskite antisolvents compared to these additives, which are applied in perovskite precursors. It means that this promising approach needs more exploration of the used materials systems, fundamentals, and engineering aspects.

In this work, amide-functionalized GQDs (AGQDs), containing amide and long hydrophobic alkyl chain groups, are used as a promising novel multifunctional group additive in the antisolvent mixture of toluene:hexane (T:H) for AAE treatment. The graded heterojunction structure reduces the concentration of the additive in perovskite film compared to the surface of perovskite. Hence, the decreased additive concentrations improve the electron transport in perovskite.^42,45,52−54^ Additionally, the formed graded heterojunction of CH_3_NH_3_PbI_3_ (MAPI):AGQDs after AAE treatment with AGQDs reduces the defect state density on the surface and grain boundaries, resulting in the facilitated charge collection at the perovskite/hole transport layer (HTL) interface and faster charge transport within the PSCs. Importantly, the hydrophobicity nature of the AGQDs used for AAE treatment and defect passivation causes a significant stability improvement on top of the device performance boosting. An enhanced PCE of 19.10% and 1.78-fold air stability are achieved after AAE treatment with AGQDs, compared with a low PCE of 16.0% for untreated devices.

## 2 Results and Discussion

### 2.1 Structural Characterization of the Graded Heterojunction PSCs

GQDs were synthesized by dispersing graphene oxide (GO) in a toluene:hexane (T:H = 2:1 ratio) solvent mixture, followed by functionalizing covalently formed GQDs with 1-dodecanamine and reducing them with glycine simultaneously.^55^ The mixture of T and H is used as an antisolvent because of two reasons: (i) the ability of these solvents for stability and dispersibility of AGQDs and (ii) T and H are two typical antisolvents for solvent engineering in the PSCs.^55−57^ As shown in Figure S1, the transmission electron microscopy (TEM) image of AGQDs (Figure S1a) displays homogeneous dots with diameters less than 20 nm and average sizes of 7 nm (Figure S1b). The characterization of AGQDs was thoroughly investigated in an earlier study.^40^ AGQDs having amide and the hydrophobic long alkyl chain (C_12_H_25_) functional groups were used as an efficient multifunctional additive in antisolvents to enhance the efficiency and stability of planar PSCs.

**Figure 1:**
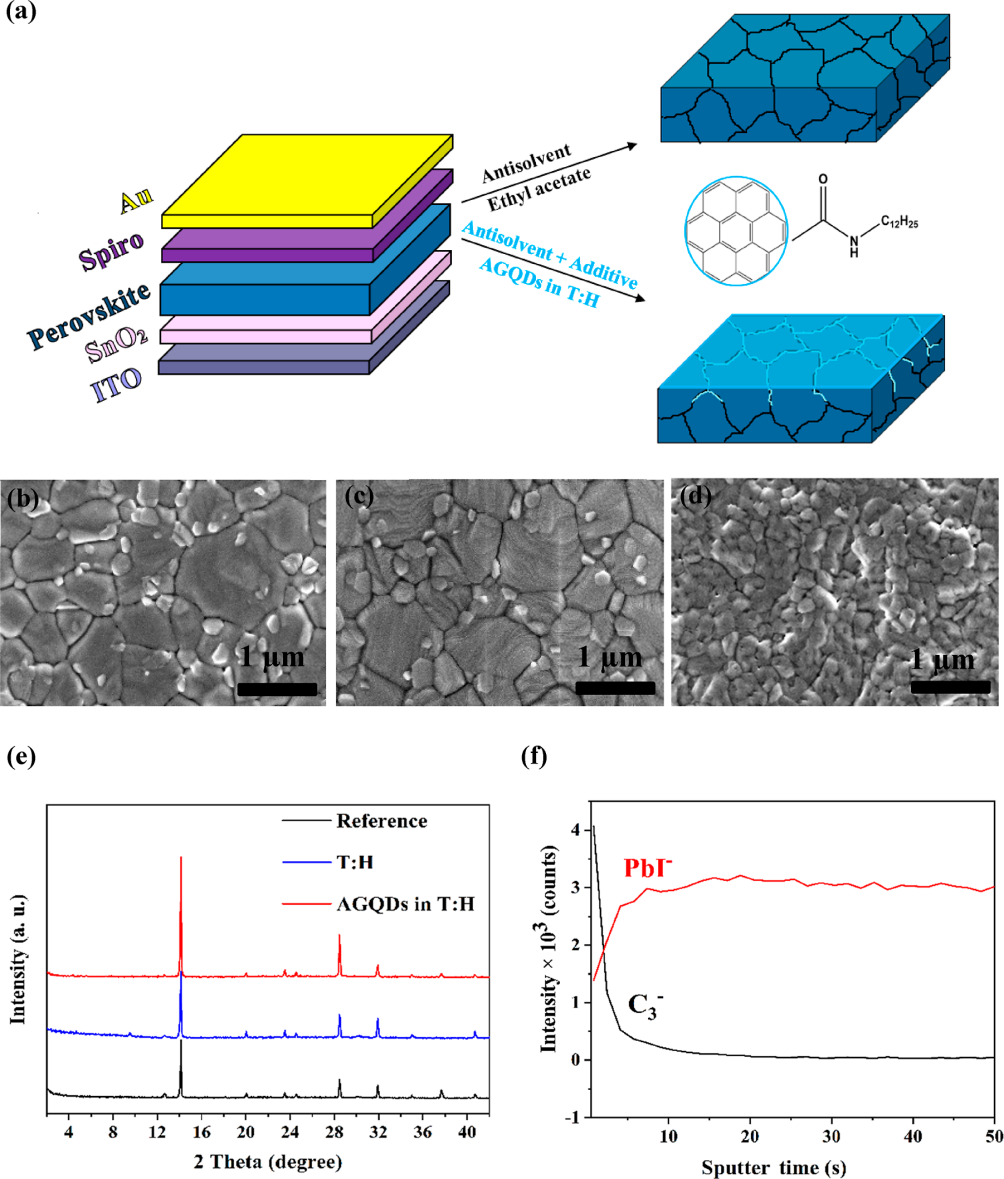
Schematic device structure and simplified chemical structure of AGQDs (a). SEM images of MAPI films with ethyl acetate antisolvent as the reference (b), with T:H mixture antisolvent (c) and with AGQDs implemented into T:H as the additive antisolvent agent (d). XRD data of MAPI films prepared with different antisolvents (e). ToF-SIMS depth profile showing PbI^–^ and C_3_^–^ components of the perovskite film with AGQDs (f).

The graded heterojunction structure was formed by dripping the solvent mixture T:H containing AGQDs as an antisolvent during the spin-coating of the perovskite precursor solution. Also, the reference was fabricated based on ethyl acetate as the most commonly antisolvent used for PSCs to clarify the effect of T and H with and without AGQDs on the performance of PSCs. Figure 1a illustrates a schematic of the resulting PSCs with a structure of indium tin oxide (ITO)/SnO_2_/MAPI:AGQDs/N2,N2,N2′,N2′,N7,N7,N7′,N7′-octakis (4-methoxyphenyl)-9,9′- spirobi[9*H*-fluorene]-2,2′,7,7′ tetramine (spiro-OMeTAD)/Au and the simplified chemical structure of AGQDs. It is expected that the dispersed AGQDs distribute mainly on the top and upper part of the perovskite and partially penetrated into the perovskite layer during crystallization as the main solvent [*N, N*-dimethylformamide (DMF), dimethyl sulfoxide (DMSO)] is extracted by the antisolvent. To confirm this, we carried out a series of verification measurements and characterization.

To investigate the effect of AAE treatment with AGQD additive on the perovskite crystallinity and the surface morphology, scanning electron microscopy (SEM), X-ray diffraction (XRD), and grazing-incidence wide-angle X-ray scattering (GIWAXS) measurements were carried out. In addition, to elucidate whether T and H antisolvent mixture offers an additional benefit in enhancing perovskite crystal quality, SEM and XRD measurements were also performed with the perovskite film prepared by T and H antisolvent without AGQD additive. Figure 1b–d shows top-view SEM images of the perovskite films with different antisolvents. It can be observed that the reference film displays clear grain boundaries with some pinholes at the surface (Figure 1b). When the T:H mixture was used as the antisolvent, the perovskite film becomes more compact and more homogeneous with fewer pinholes (Figure 1c). In addition, it can be clearly seen that the grains are coarsened and become larger and denser after adding T:H mixture as the antisolvent. Figure S2a,b shows the histograms of grain size distributions in perovskite films treated with ethyl acetate and T:H antisolvents. The film treated with T:H mixture antisolvent shows distinctly a grain-size distribution shifted toward larger values compared to the one treated with ethyl acetate. Interestingly, after adding AGQDs into T:H antisolvent, the grain texture is completely changed on the top surface and an ultrathin layer is formed on top of the perovskite film, which provides more contact between perovskite and HTL (Figure 1d). As shown in the cross-sectional SEM images of MAPI without and with AGQDs in Figure S3, the incorporation of the AGQDs increases the thickness of the resulting graded heterojunction perovskite film. It can be attributed to the promoted crystal growth by AAE with AGQDs which makes the grains in perovskite film larger and therefore increases the thickness of the perovskite film slightly. In addition, more compact grains and fewer grain boundaries of the perovskite film can be obtained after adding AGQD additive, which is consistent with the top-view SEM images. However, in the cross-sectional SEM image, no GQD film at the graded heterojunction perovskite surface is visible probably due to its ultrathin thickness.

Figure 1e shows the XRD patterns of the obtained MAPI films with and without AAE treatment on ITO substrates. All samples exhibit the same characteristic Bragg peaks of a tetragonal MAPI film at 14, 24, 28, and 32° assigned to (110), (202), (220), and (310) planes, respectively. The intensity of MAPI with AGQD diffraction peaks is much stronger than the reference, which verifies the enhanced crystallinity of the MAPI film after AAE treatment with AGQDs. In addition, the absence of additional peaks and peak shifts demonstrates that the MAPI film remains in the tetragonal structure after the AAE treatment, and AGQDs are distributed just on the surface and at the grain boundaries of the perovskite film. This evidence confirms that AAE with AGQD additive can affect the perovskite kinetics and subsequently provide high-quality perovskite films, likely due to two main reasons: (i) the antisolvent mixture of T and H promotes the formation of perovskite crystal seeds and enlarges the perovskite grains, resulting in a dense and compact morphology,^13,57^ (ii) the implemented AGQDs into the antisolvent slow down the rate of perovskite crystallization and boost the crystal growth with promoted order by interactions with the perovskite (between C=O and Pb^2+^).^49,58^

The 2D GIWAXS measurements for the reference and the perovskite with AGQD films are shown in Figure S4. Both samples exhibit identical Bragg reflections for the perovskite film with a stronger peak at *q* = 1.0 °A^–1^ assigned to the (110)/(002) lattice of the tetragonal indexed MAPI perovskite (space group *I*4*cm*). The corresponding pseudo-XRD patterns of both films represent the same diffraction peaks (Figure S5). These results prove that the crystallinity of the 3D perovskite film remains without any significant change in lattice spacing or texture after AAE treatment with AGQDs.

The time-of-flight secondary ion mass spectrometry (ToF-SIMS) depth profile was measured to study the depth profile of AGQDs in the graded heterojunction perovskite film. C_3_^–^ and PbI^–^ were used as an indicator of AGQDs and perovskite, respectively. As shown in Figure 1f, the depth profile of AGQDs exhibits a steep decline with a spatial overlap on the PbI^–^ profile. This evidence confirms that AAE with AGQDs can form the graded heterojunction structure of MAPI:AGQDs with a graded distribution of AGQDs near the interface. According to the unchanged XRD and the size mismatched between AGQDs and perovskite elements, the AGQDs are mainly accumulated at the grain boundaries of the perovskite film.

### 2.2 Photovoltaic Performance of the Graded Heterojunction PSCs

**Figure 2:**
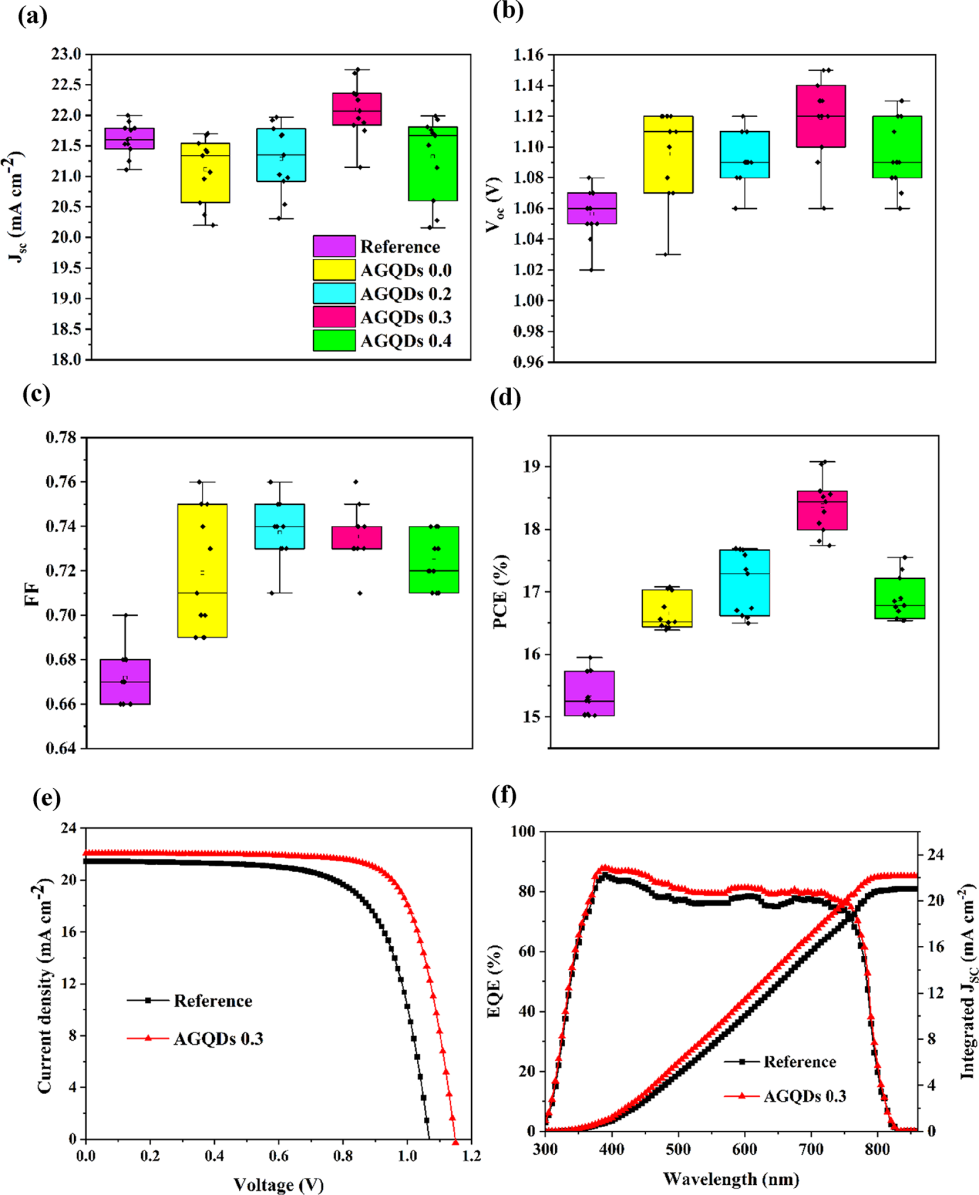
Statistical distribution of photovoltaic parameters (from 11 individual solar cells each) after AAE treatment with various amounts of AGQDs: *J*_sc_ (a), *V*_oc_ (b), *FF* (c), and PCE (d). *J*–*V* curves of reference and AGQDs 0.3 PSCs under AM 1.5G illumination with the light intensity of 100 mW cm^–2^ (e). EQE spectra of reference and AGQDs 0.3 PSCs (f).

To well understand the effect of antisolvent engineering with and without solid additives as well as the amount of additive on the PSC photovoltaic performance, PSCs were fabricated with various amounts of AGQDs in T:H as antisolvents (0.0, 0.2, 0.3, and 0.4 mL denoted as AGQDs 0.0, AGQDs 0.2, AGQDs 0.3, and AGQDs 0.4, respectively). The statistical distribution of the photovoltaic parameters from 11 individual solar cells fabricated in different experimental batches for each sample is summarized in Figure 2a–d. Moreover, the corresponding photovoltaic performance average values are listed and summarized in Table S1.

As shown in Figure 2a–d, all the PSCs based on T:H antisolvent engineering without and with AGQDs represented better efficiencies compared to the reference based on ethyl acetate antisolvent. Significantly, the AAE-treated PSCs with various amounts of AGQD additives achieved higher average PCEs in comparison with those fabricated based on T:H antisolvent without AGQDs. The average PCE increased from 16.66 to 18.37%, when the amount of AGQD antisolvent increased from 0.0 to 0.3 mL. However, a further increase of AGQDs from 0.3 to 0.4 mL exhibited a PCE drop down from 18.37 to 16.88%. It can be due to the fact that ongoing addition of AGQDs can block the surface of perovskite film and prevent efficient charge transfer at the interface. Altogether, the best photovoltaic performance was obtained by applying AAE treatment with 0.3 mL of AGQD additive. Therefore, further measurements were carried out on PSCs based on AGQDs with the optimum amount of 0.3 mL (AGQDs 0.3) unless otherwise stated.

Figure 2e shows the current density–voltage (*J*–*V*) curves of the champion reference and AGQDs 0.3 solar cells under AM 1.5G illumination with the light intensity of 100 mW cm^–2^. The reference cell exhibited a PCE of 16.0% with a *V*_oc_ of 1.07 V, *J*_sc_ of 21.45 mA cm^–2^, and *FF* of 0.70. The AGQDs 0.3 device showed a noticeably improved performance of about 20%, with a PCE of 19.10%, *V*_oc_ of 1.15 V, *J*_sc_ of 22.07 mA cm^–2^, and *FF* of 0.76. The enhanced PCE is related to all the improved photovoltaic performances, indicating the effective role of AAE treatment with AGQDs and formed graded heterojunction structures in PSCs.

The enhanced photovoltaic parameters are also verified by the external quantum efficiency (EQE) measurements displayed in Figure 2f. As expected, the EQE spectrum of the AGQDs 0.3 device exhibited a superior incident photon-to-current conversion efficiency to the reference device in the wavelength range between 360 to 860 nm, and the integrated *J*_sc_ obtained by EQE is 21.04 and 22.14 mA cm^–2^ for reference and AGQDs 0.3 PSCs, respectively, which is well-matched with the *J*_sc_ from *J*–*V* curves measurements.

### 2.3 Defect Passivation and Charge-Transfer Mechanisms of the Graded Heterojunction PSCs

**Figure 3:**
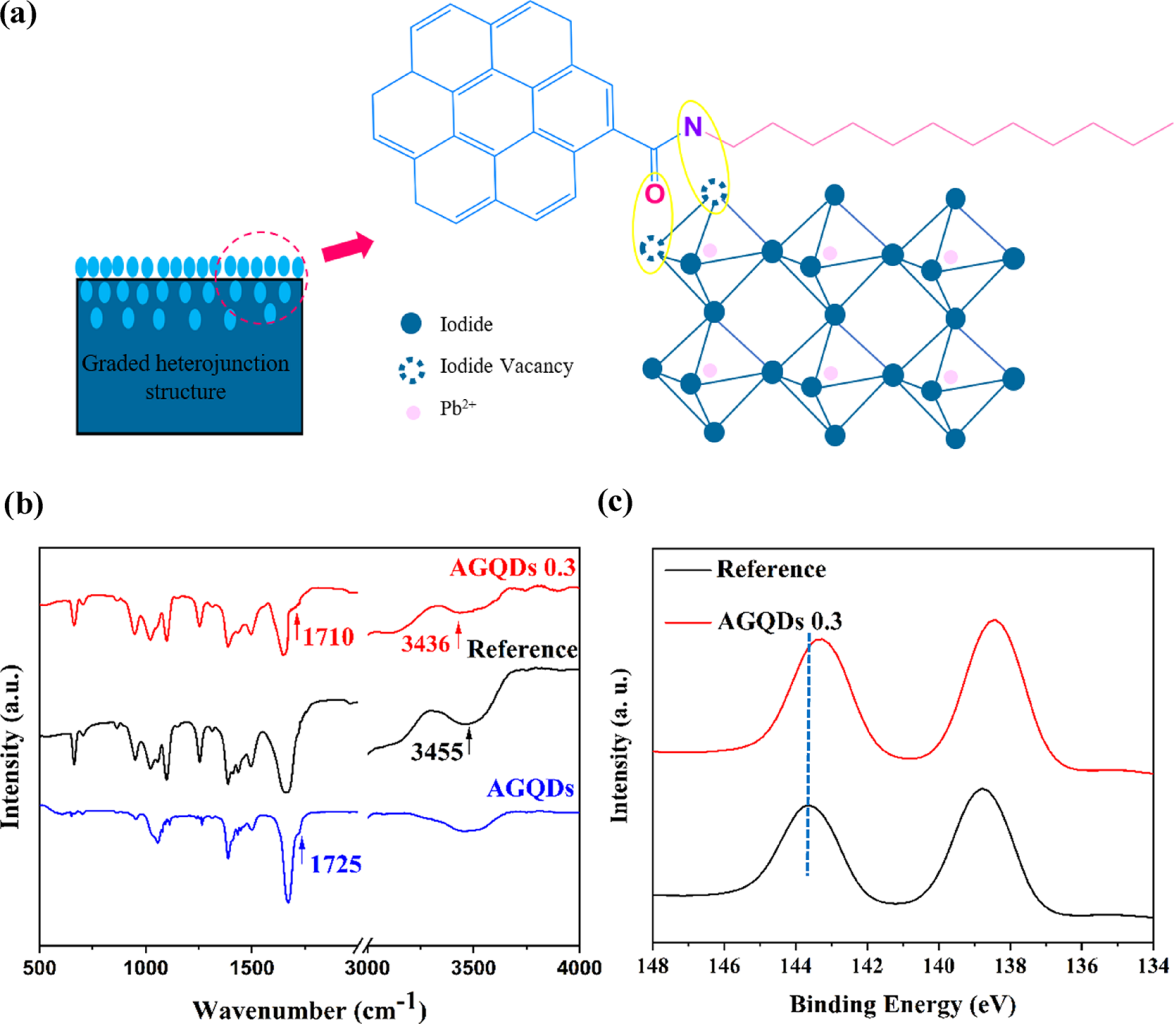
Schematic of passivation mechanism of the perovskite film by the graded heterojunction structure after AAE treatment with AGQDs (a). FTIR spectra of AGQD, reference, and AGQDs 0.3 perovskite films (b). XPS spectra of reference and AGQDs 0.3 perovskite films (c).

For gaining a deeper understanding of efficiency improvement due to AAE treatment with AGQD additive, a series of advanced characterizations were carried out. As shown in Figure 3a, the interaction between the functional groups of AGQDs and the uncoordinated Pb^2+^ ions can potentially passivate the surface and grain boundaries trap states in perovskite film. This important impact was confirmed by Fourier transform infrared spectroscopy (FTIR) and X-ray photoelectron spectroscopy (XPS) measurements.

Figure 3b displays the FTIR spectra of AGQDs and perovskite film without and with AGQDs. For the AGQDs, the stretching vibration band of C=O can be observed at 1725 cm^–1^, while the C=O vibration was moved to 1710 cm^–1^ in perovskite film with AGQDs (AGQDs 0.3), confirming the interaction between C=O and uncoordinated Pb^2+^ ions of perovskite. Furthermore, the band centered at 3500–3300 cm^–1^ is attributed to the stretching vibration band of N–H in-plane stretching.^59,60^ The stretching vibration peak of N–H at 3455 cm^–1^ in perovskite film shifts to 3436 cm^–1^ in modified perovskite by AGQDs, which demonstrates not only C=O but also N atoms of 1-dodecanamine of AGQDs coordinated with Pb^2+^ ions of perovskite.

To investigate the compositional and chemical changes on the surface of the perovskite film after adding AGQDs, XPS analysis of Pb 4f was conducted. As shown in Figure 3c, in the graded heterojunction AGQDs 0.3 film, the pair peaks of Pb (4f_7/2_ and 4f_5/2_) exhibit a red shift compared to those in the reference film, demonstrating that there is a chemical interaction between the perovskite material and AGQDs. This evidence confirms that the AGQDs passivate the uncoordinated Pb^2+^ ions via anchoring to the perovskite by amide functional groups.

UV–visible measurements were carried out to examine the effect of AAE treatment with AGQDs on the optical property of the perovskite films. Figure S6 shows the UV–visible spectra of perovskite films with and without AGQDs. The treated film displays a stronger absorption intensity at 400–500 nm than the reference, which can be attributed to the improved crystal quality as well as the slightly different thickness of the perovskite layer after AAE treatment and forming of graded heterojunction MAPI:AGQD structures. These characteristics are very beneficial to provide higher *J*_sc_ in PSCs with AAE treatment by AGQDs.

**Figure 4:**
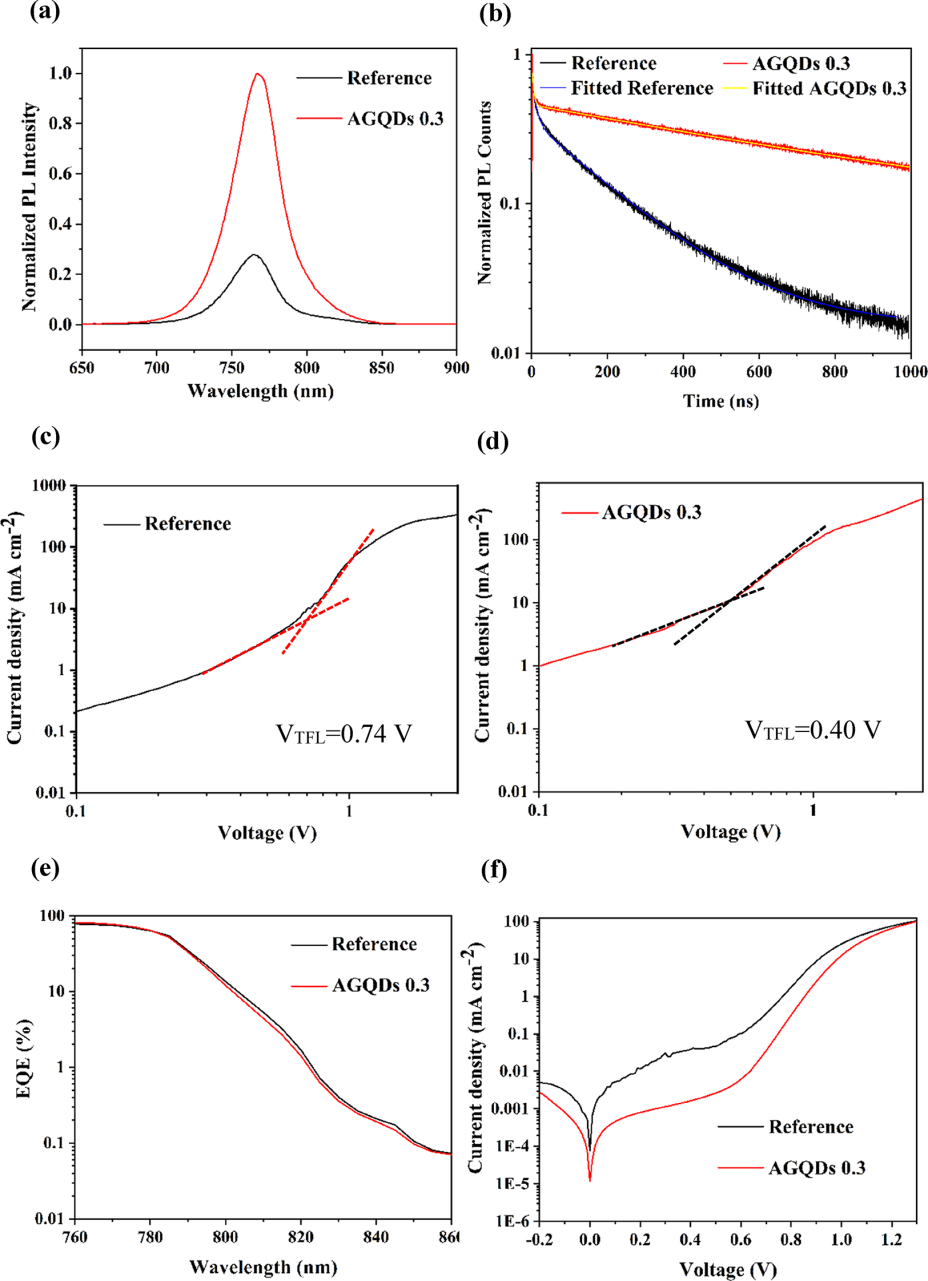
Normalized PL spectra of the reference and AGQDs 0.3 perovskite films deposited on a glass substrate (a). TRPL decay spectra of the reference and AGQDs 0.3 perovskite films (b). Dark *J*–*V* curves of the reference electron only device (c). Dark *J*–*V* curves of the AGQDs 0.3 electron only devices (d). EQE of the reference and AGQDs 0.3 PSCs (e). Dark *J*–*V* curves of the reference and AGQDs 0.3 PSCs (f).

Photoluminescence (PL) and time-resolved photoluminescence (TRPL) were conducted to study the charge carrier lifetime of the perovskite films with and without AAE by AGQD additive (Figure 4a,b). As shown in Figure 4a, the PL intensity significantly increases in the presence of AGQDs at 770 nm, indicating the remarkably suppressed non-radiative recombination in AGQD passivated perovskite film. This specification has a significant improving effect on *FF* and *V*_oc_.

Furthermore, TRPL decay curves of reference and treated AGQD films were fitted through the dual exponential functions (eq 1) to calculate the sample carrier
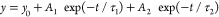
1

The average lifetime of perovskite film with AGQDs considerably increases to 486 ns, which is 4.5 times longer than the one from the reference (108 ns). These results imply a lower trap state density and a longer-lived charge carrier as a result of defect passivation at the surface and grain boundaries of graded heterojunction perovskite film after AAE treatment with AGQD additive.

The charge extraction behavior at the perovskite/HTL interface was studied by steady-state PL measurements on glass/MAPI (without and with AGQDs)/HTL. As shown in Figure S7, the PL signal of the perovskite with AGQDs is quenched more effectively than the reference. The collected information highlights the ability of AGQDs in boosting charge extraction and suppressing the charge recombination due to high carrier mobility and conductive nature of GQD skeleton and defect passivation by their functional groups across the MAPI/HTL interface.

To further confirm the trap passivation via AAE treatment with AGQD additive, the space charge limited current analyses were performed with the electron-only device structure of ITO/SnO_2_/MAPI (with or without AGQDs)/phenyl-C61-butyric acid methyl ester (PCBM)/Au. The linear region is attributed to the Ohmic response at the low bias. The current rapidly increases, when the voltage increases above the kink point, indicating that the trap states are completely filled by the injected carrier. The trap density (*N*_t_) was calculated via trap filled limit voltage (*V*_TFL_), referring to the applied voltage at the kink point, using eq 2(61)
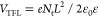
2where *e* is the elementary charge, ε_0_ is the vacuum dielectric constant, ε is the relative dielectric constant, and *L* is the thickness of the MAPI layer. As depicted in Figure 4c,d, the *V*_TFL_ values of 0.74 and 0.40 V and *N*_t_ values of 4.7 × 10^15^ and 2.14 × 10^15^ cm^–3^ are extracted for reference and AGQDs 0.3 devices, respectively. This observation further confirms that the graded heterojunction structure is composed of AAE with AGQDs and reduces the trap states in the perovskite film.

Moreover, EQE was used to study sub-gap states after the passivation of defects via AAE with AGQDs. The sub-gap states are revealed in the EQE spectrum at a wavelength above 800 nm. As shown in Figure 4e, the sample treated with AGQDs displays a lower photocurrent signal than the reference in the sub-gap region from 800 to 860 nm. It indicates that AAE with AGQDs reduces effectively the non-radiative recombination originated from sub-gap states and increases the *V*_oc_ and consequently the photovoltaic performance.

Dark current was measured to investigate the effect of AAE treatment on the carrier recombination kinetics. The dark *J*–*V* curves of the reference and AGQDs 0.3 devices are displayed in Figure 4f. The ideality factor could be calculated from the slope of the natural logarithm of the dark current (*J*_d_) against the voltage according to the equation below^62^
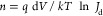
3where *n* is the ideality factor, *q* is the elementary charge, *k* is the Boltzmann constant, and *T* is the temperature. The ideality factors of 1.69 and 1.26 are calculated for the reference and AGQDs 0.3 samples, respectively. It suggests that non-radiative recombination was significantly suppressed by AAE treatment by AGQDs. These results are in total agreement with enhanced *V*_oc_ and *FF*.

### 2.4 Hydrophobicity and Stability of the Graded Heterojunction PSCs

**Figure 5:**
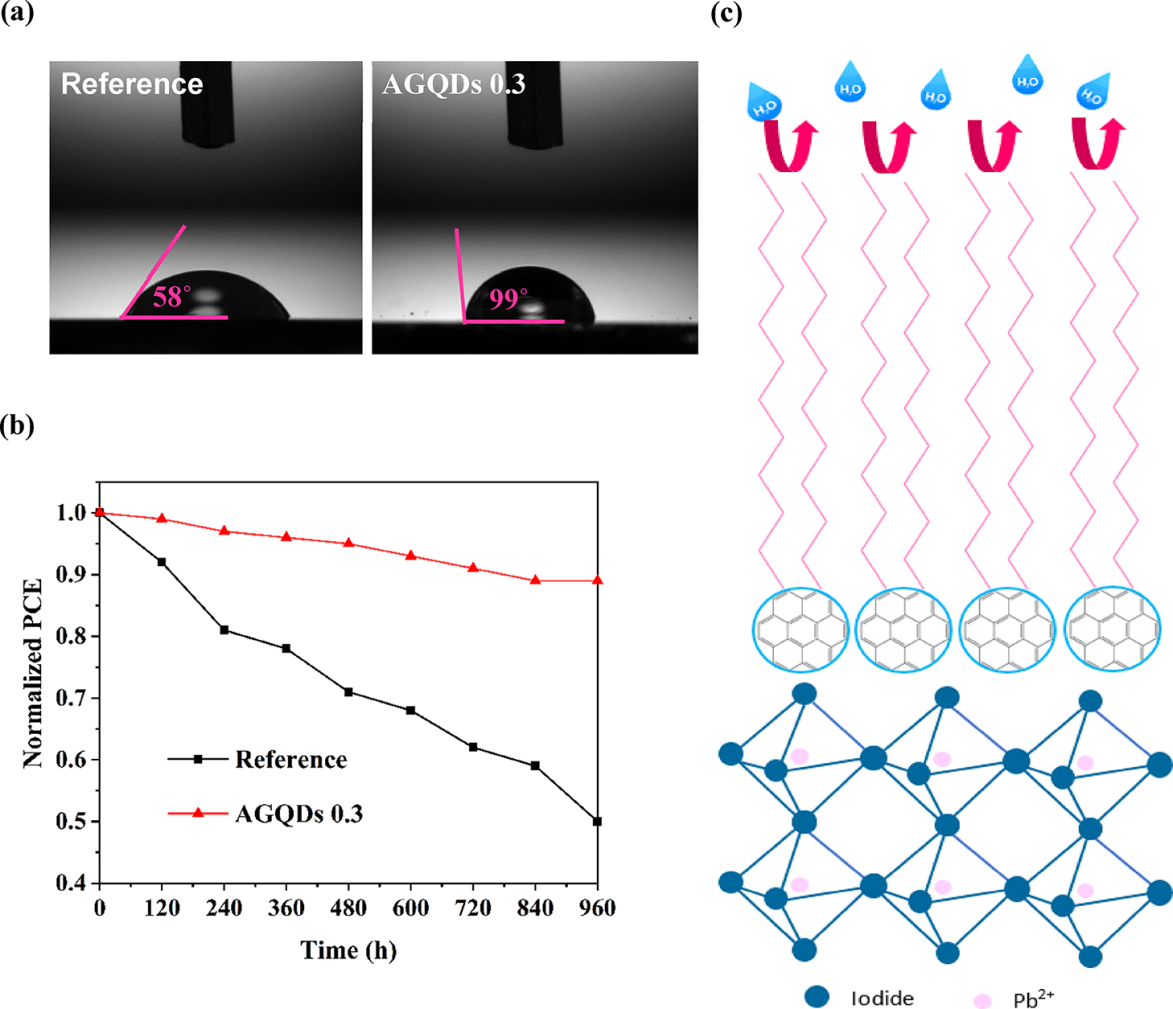
Water contact angles of the reference and AGQDs 0.3 perovskite films (a). Normalized PCE of the reference and AGQDs 0.3 PSCs in ambient conditions without encapsulation (b). Schematic of the moisture protection mechanism of the perovskite film after AAE treatment by AGQDs (c).

To investigate the protective effect of the hydrophobic alkyl chain of AGQDs on the perovskite film surface, the contact angle measurement of a water droplet was carried out. Figure 5a displays the contact angles of 58 and 99° for perovskite film without and with AAE treatment with AGQDs, respectively. It indicates that AGQDs enhance the hydrophobicity of perovskite film owing to the long alkyl chain (C_12_H_25_) groups on their surface.

Finally, the effect of AAE treatment with AGQDs on the PSC stability was studied under the ambient conditions without any encapsulation. As shown in Figure 5b, the reference PSC demonstrates 50% decay of its initial PCE over 960 h, while the AGQD PSC retains 89% of its initial PCE. The dramatically improved moisture stability after AAE treatment can be related to two main reasons: (i) while the AGQDs anchor to the perovskite film by amide side, the exposed hydrophobic long alkyl chain (C_12_H_25_) groups provide a moisture barrier on the top of the perovskite film (Figure 5c). (ii) The high-quality perovskite film without a pinhole and a reduced trap state density at the surface and grain boundaries due to AGQD passivation protect the perovskite film from moisture and oxygen penetration.

Our findings indicate that the one-step AAE method with AGQDs is a very promising route for boosting the efficiency and stability of PSCs. More importantly, the same additive via various methods proves different benefits for improving PSCs. In our previous work, AGQDs were used as just a passivator between perovskite and HTL by interface engineering method.^40^ Comparing with the interface engineering, AAE route by AGQDs is more efficient and effective for boosting the efficiency because of two main reasons: (i) AGQDs added by AAE effectively control perovskite crystallization by the mixture antisolvent (T:H) as well as interactions between C=O of AGQDs and perovskite, while AGQDs added by interface engineering only affect the surface crystallization. (ii) Implementation AGQDs via AAE inhibits the radiative and non-radiative charge recombination by passivating defects at the interface, grain boundaries, and also the upper part of the perovskite film through the formation of graded heterojunction structures. However, interface engineering reduces defects only at the surface and grain boundaries of perovskite film. As a result, AAE method by AGQDs leads to a higher device performance than interface engineering method (a PCE of 19.10% compared to 18.30%). Moreover, AAE method enhances the moisture stability about 12% more than interface engineering. This evidence is related to the ability of AAE to first uniformly distribute AGQDs on the surface and top of perovskite film, which lead to a more effective moisture protection. Second, AGQDs introduced by AAE reveal more effective defect passivation than interface engineering, which results in more stability.

## 3 Conclusions

In conclusion, we investigated an effective AAE strategy to improve the photovoltaic performance and moisture stability of PSCs simultaneously. AGQDs, a promising novel multifunctional group additive with carbonyl, amine, and long hydrophobic alkyl chain groups, was introduced in T and H (2:1 ratio) mixture antisolvent for AAE. ToF-SIMS and XRD results indicate that this method could form a mixed heterojunction interlayer of AGQDs. The multi-beneficial approach of AAE with AGQDs enables (i) enhancing the crystallinity of the perovskite film, (ii) passivating defects and trap states at the surface and grain boundaries, (iii) improving the charge transfer between the perovskite film and HTL, and (iv) increase the moisture stability of PSCs. By applying AAE with AGQDs, a significant performance improvement of around 20% was achieved. Moreover, the AAE-treated unencapsulated PSC retains around 90% of its initial PCE after 960 h. Our strategy of AAE with multi-functional AGQDs provides a cost-efficient promising additive and conventional approach to fabricate highly efficient and stable PSCs.

## 4 Experimental Section

### 4.1 Materials

Tin (IV) oxide (SnO_2_, 15% in H_2_O colloidal dispersion) was purchased from Alfa Aesar. The MAPbI_3_ perovskite precursors, lead (II) iodide (PbI_2_, 99%) and methylene ammonium iodide (MAI, 97%), were purchased from TCI. N2,N2,N2′,N2′,N7,N7,N7′,N7′-octakis (4-methoxyphenyl)-9,9′- spirobi[9*H*-fluorene]-2,2′,7,7′ tetramine (spiro-OMeTAD, 99%) were all purchased from Borun Chem. Exfoliated GO, dodecyl amine (DDA, ≥ 99%), glycine (≥99%), anhydrous DMF (99.8%), anhydrous DMSO (≥ 99.5%), anhydrous ethyl acetate (99.8%), anhydrous acetonitrile (99.8%), anhydrous toluene (99.8%), anhydrous chlorobenzene (99.8%), and anhydrous hexane (95%) were purchased from Sigma-Aldrich Company. All the chemicals and reagents are directly used without any further purification.

### 4.2 Synthesis of AGQDs

Exfoliated GO was used as a starting material to synthesize HGQDs according to the literature-described process.^55^ After ultra-sonication of 0.002 g of GO in 40 mL of toluene, another dispersion of 0.001 g of DDA in 20 mL of hexane and 0.0003 g of glycine was mixed. This mixture was stirred mechanically for 40 min. Then, it was refluxed at 120 °C for 10 h and subsequently centrifuged at 7000 rpm for 7 min. The supernatant dispersion, HGQDs containing amino-alkyl chains (NHC_12_H_25_), were obtained for further use and characterization.

### 4.3 Device Fabrication

The PSCs were fabricated based on ITO/SnO_2_/MAPI:AGQDs/spiro-OMeTAD/Au structure. First, the ITO :glasses were etched by Zn powder and HCl solution, followed by washing process in each of the following individually: Helmanex solution, deionized water, acetone, and ethanol in an ultrasonic bath for 15 min. Then, oxygen plasma was used to clean the substrates for 15 min. The SnO_2_ layer was deposited on ITO via spin coating of 2.67% SnO_2_ solution at 4000 rpm for 35 s as an electron transport layer, followed by annealing at 150° C for 30 min. The treated substrates by oxygen plasma were transferred to the glovebox with an N_2_-controlled atmosphere to complete the fabrication process. Perovskite solution was prepared by dissolving PbI_2_ (775 mg), MAI (254 mg M), and DMSO (114 μL) in 1 mL of DMF under stirring at 70 °C for 30 min. The perovskite solution was spin-coated by an antisolvent-assisted two-step procedure: first at 1000 rpm for 30 s and then at 3500 rpm for 20 s. In the middle of the second step, different amounts (200, 300, and 400 μL) of the toluene (T) and hexane (H) mixture antisolvent with 2:1 ratio containing AGQDs were dropped on top of the perovskite film under spinning. Then, the perovskite films were annealed at 130 °C for 10 min. The reference samples were fabricated with ethyl acetate as the most commonly used antisolvent for PSCs. HTL was prepared by mixing 72.3 mg of spiro-OMeTAD and 35 μL of lithium bis(trifluoromethanesulfonyl)imide (LiTFSI) solution (520 mg in 1 mL of acetonitrile) with 17.5 μL of 4-tert-butylpyridine (4- TBP) in 1 mL of chlorobenzene, and then 80 μL of the mixture solution was spin-coated on top of the perovskite film at 3000 rpm for 30 s. Afterward, the samples were transferred into a desiccator overnight. Finally, 70 nm of Au as the counter electrode was vacuum-evaporated on top using a metal mask.

### 4.4 Characterization Methods

SEM images were recorded using an FEI Helios G3 UC instrument equipped with a secondary electron detector at an acceleration voltage of 2 kV for the top-view images and 5 kV for the cross-sectional images. Cross-sectional images were performed through a backscatter electron detector, and top-view images were recorded using both a backscatter electron detector and secondary electron through-the-lens detector. TEM was performed in the annular dark field mode on a probe-corrected FEI Titan Themis at 300 kV. XPS analyses were conducted using a non-monochromized X-ray source, VSW TA10 Mg-Kα radiation, and a VSW HA100 electron analyzer. The peak of I3 d_5/2_ was calibrated at a binding energy of 619.3 eV to eliminate the peak shift because of the charging effect. A convolution of a Doniach–Šunjićfunction and a Gaussian function with linear background subtraction was applied to fit XPS peak components. XRD analysis was carried out using a Bruker D8 Discover diffractometer with Ni-filtered Cu Kα_1_-radiation (λ = 1.5406 Å) and a position-sensitive semiconductor detector (LynxEye). GIWAXS measurements were conducted using a Pilatus 300k detector (Dectris) at an energy of 12.49 keV (X-ray wavelength 0.99 Å) at the P03 beamline at DESY, Germany.^63^ The incidence angle was set between 0.1 and 0.8° with a sample–detector distance of 180 mm. 2D-GIWAXS data were corrected for path attenuation, detector absorption, photon polarization, solid angle, and flat field using the software GIXSGUI.^64^ Pseudo-XRD patterns were created by radial integration of the available *q*-range of the 2D GIWAXS images to index the Bragg rings. X-ray powder diffraction patterns for analyzing pseudo-XRD data were simulated by the software VESTA.^65^ UV–visible spectra were measured using a Perkin Elmer Lambda 1050 spectrophotometer with an integrated sphere. All PL measurements were obtained using a Picoquant Fluotime 300 spectrofluorometer (PicoQuant GmbH) in air. The samples were excited by a pulsed solid-state laser of 375 nm wavelength (LDH375, PicoQuant). The *J*–*V* curves were recorded using a Newport OrielSol 2A solar simulator with a Keithley 2400 source meter under simulated AM 1.5G sunlight, calibrated to 100 mW cm^–2^ with a Fraunhofer ISE certified silicon cell (KG5-filtered). The active area of PSCs was defined by a metal aperture mask of 0.0831 cm^2^. *J*–*V* curves were carried out using scanning the input bias from 0 to 1.5 V (forward scan) at a scan rate of 0.1 V/s. For EQE measurements, the respective solar cell was illuminated with chopped monochromatic light of a tungsten light source. To obtain the incident illumination power to calculate EQE (λ), the response of a reference diode was used. The resulting current response was recorded using a lock-in amplifier (signal recovery 7265 Stanford Research Systems 830) at a chopping frequency of 14 Hz. The theoretical short-circuit current was extracted by integrating the resulting EQE curves over the reference solar spectral irradiance under one sun condition. The depth profiles were measured with a TOF-SIMS setup from ION-TOF GmbH (type: “TOF-SIMS 5”). Pulsed Bi^3+^ primary ions from a 30 keV liquid-metal ion gun were used as an analytical source, and a 500 eV Cs^+^ source was utilized as a sputtering ion source. The TOF-SIMS depth analysis was performed on a 100 × 100 μm^2^ area in the so-called spectrometry mode inside a 300 × 300 μm^2^ sputtering crater.

## Supporting Information Available

TEM image, size distribution measurement, size distribution histogram, cross-sectional SEM images, 2D GIWAXS and pseudo-XRD data, PL measurements, UV–visible absorption measurements, and device performance parameters (PDF)
