# Supplementary material for: Antisolvent Additive Engineering for Boosting Performance and Stability of Graded Heterojunction Perovskite Solar Cells Using Amide-Functionalized Graphene Quantum Dots
Source: ACS Appl Mater Interfaces. 2022 Nov 29;14(49):54623–34. doi: 10.1021/acsami.2c12944 (PMC9756295; doi:10.1021/acsami.2c12944)
Supplement: Supplementary file 1 — am2c12944_si_001.pdf [file am2c12944_si_001.pdf]

Supporting Information

Antisolvent Additive Engineering for Boosting Performance and Stability of Graded Heterojunction Perovskite Solar Cells Using Amide-Functionalized Graphene Quantum Dots

Elahe Khorshidi [a,b], Behzad Rezaei [b], Arash Kavousighahfarokhi [c], Jonas Hanisch [d], Manuel A. Reus [e], Peter Müller-Buschbaum [e,f], and Tayebeh Ameri [a,g]**

[a] Department of Chemistry and Center for NanoScience (CeNS), Ludwig-Maximilians-Universität München, Butenandtstrasse 5-13 (E), 81377 Munich, Germany

[b] Department of Chemistry, Isfahan University of Technology, Isfahan 84156–83111, Iran

[c] Department of Electrical and Electronic Engineering, Faculty of Engineering, Universiti Putra Malaysia, 43400 UPM Serdang, Selangor Darul ehsan, Malaysia

[d] Zentrum für Sonnenenergie- und Wasserstoff-Forschung Baden-Württemberg (ZSW), Meitnerstraße 1, 70563 Stuttgart, Germany

[e] Lehrstuhl für Funktionelle Materialien, Physik-Department, Technische Universität München, James-Franck-Straße 1, 85748 Garching, Germany

[f] Heinz Maier-Leibnitz Zentrum (MLZ), Technische Universität München, Lichtenbergstr. 1, 85748 Garching, Germany

[g] Institute for Materials and Processes, School of Engineering, University of Edinburgh, Sanderson Building, Robert Stevenson Road, EH9 3FB Edinburgh, UK

*Email: e.khorshidi@ch.iut.ac.ir

*Email: tayebeh.ameri@ed.ac.uk

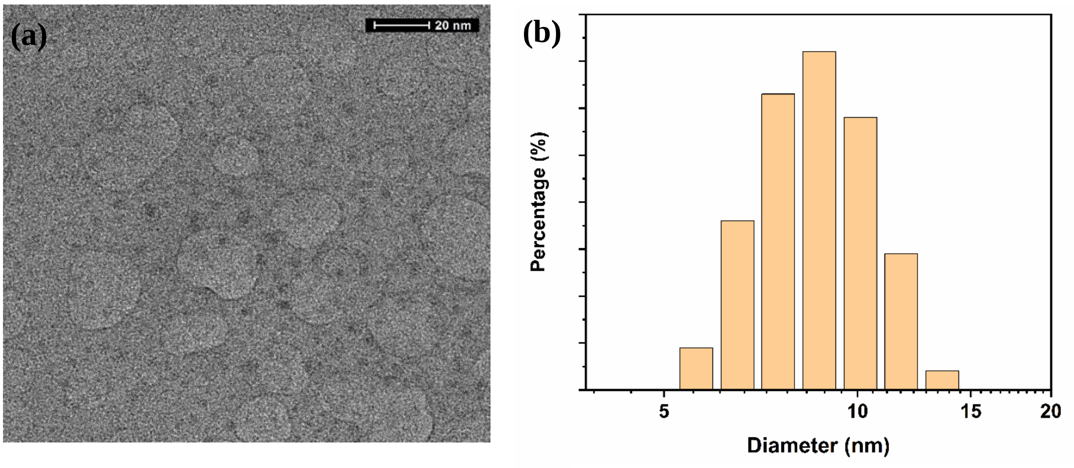

Figure S1. TEM image of AGQDs (a) and size distribution of AGQDs (b).

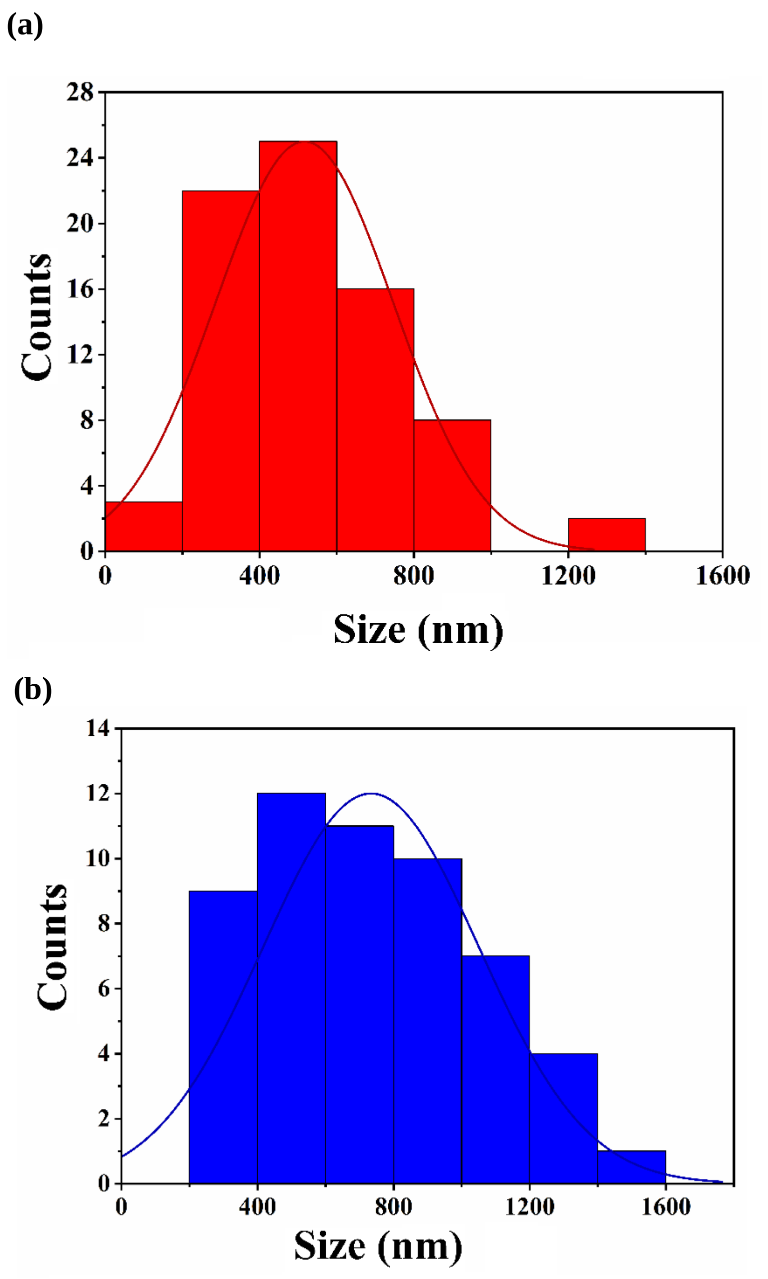

Figure S2. Histograms of grain size distributions in perovskite films treated with ethyl acetate (a) and T:H (b) antisolvents.

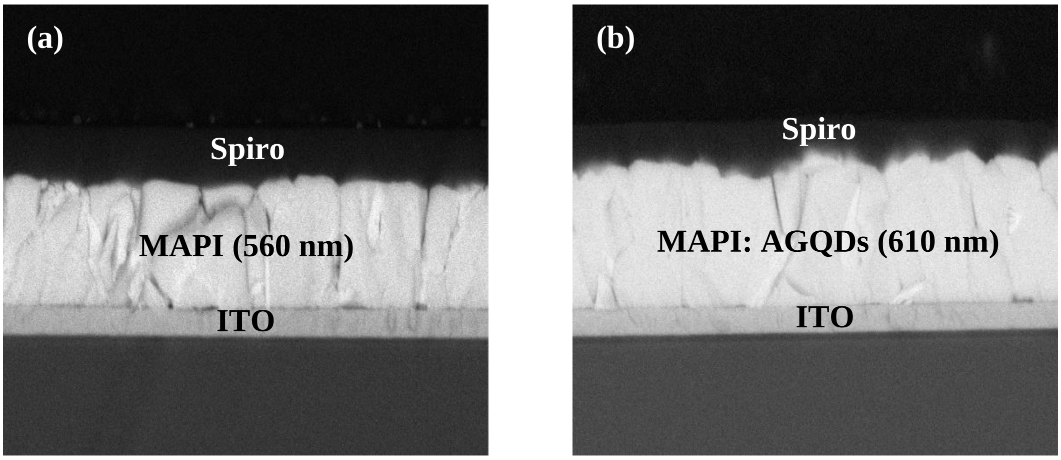

Figure S3. Cross-sectional SEM images of reference (a), and AGQDs 0.3 (b) PSCs.

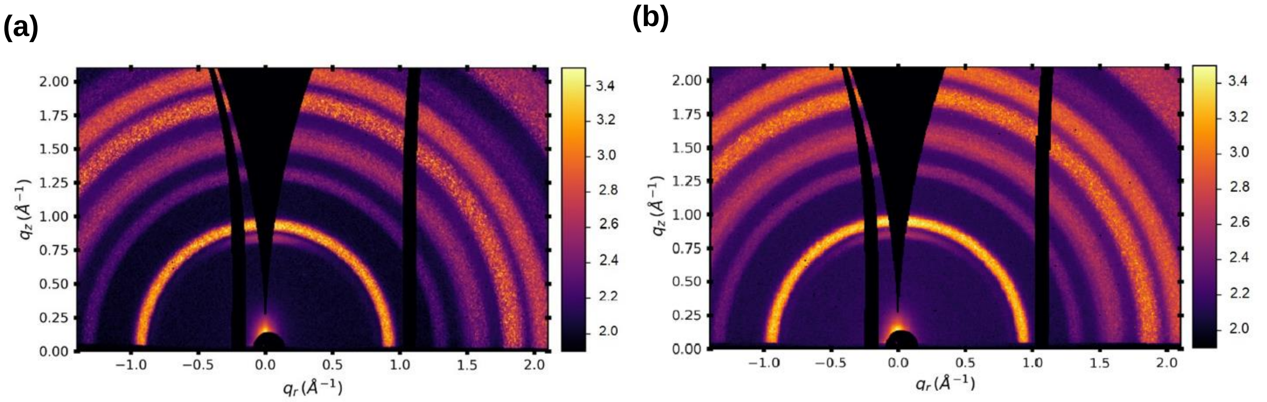

Figure S4. 2D GIWAXS data of reference (a) and AGQDs 0.3 perovskite films (b) at an incident angle of 0.26º.

(a)

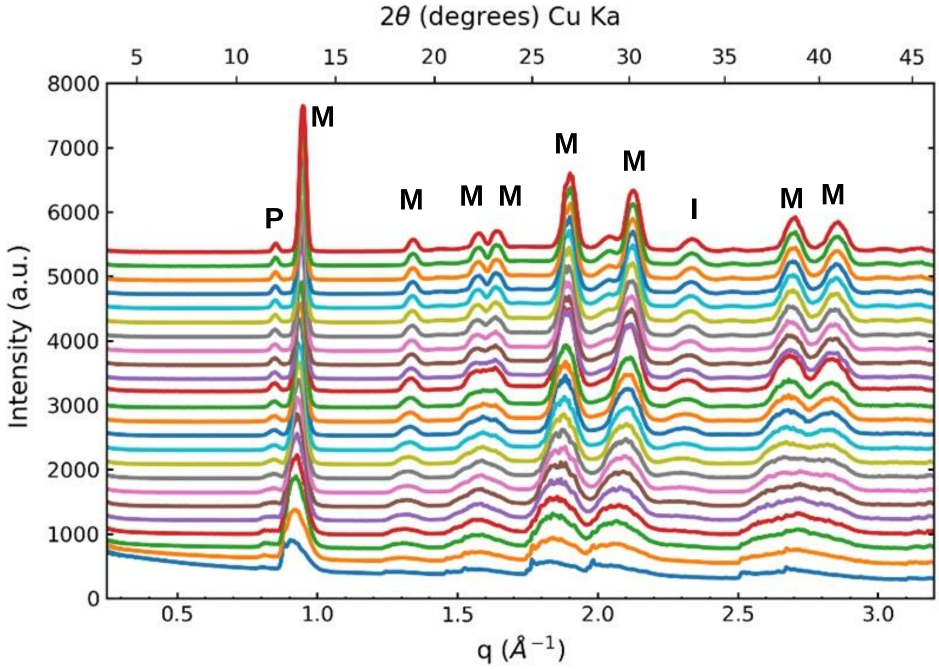

(b)

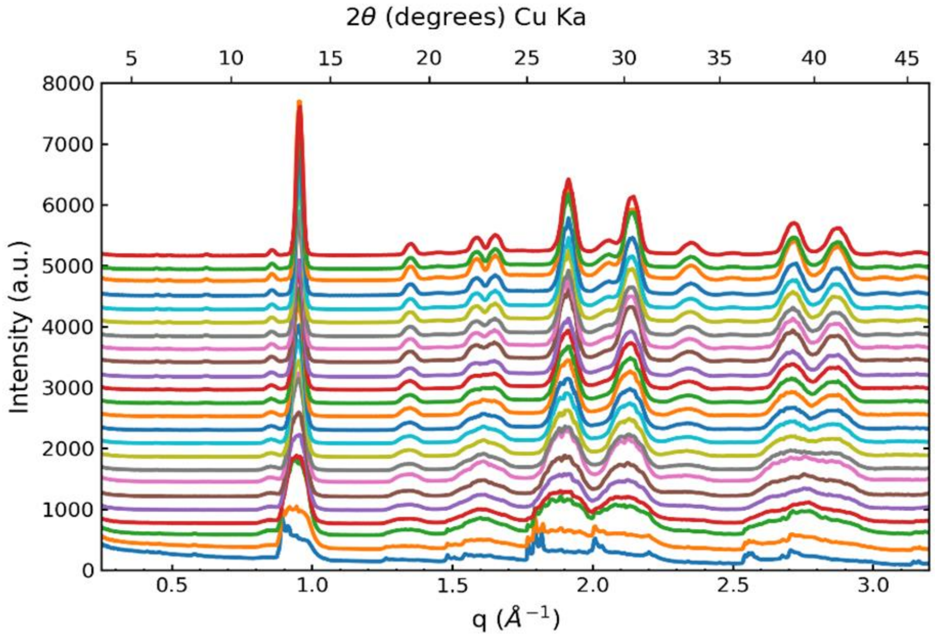

Figure S5. Pseudo-XRD data (radial cake cuts) extracted from 2D GIWAXS data, taken at different incidence angles. Incident angle increase from bottom to top from 0.14º to 0.6º of reference (a) and AGQDs 0.3 (b) films (**M**: $MAPbI_3$; **P**: PbI_2; **I**: ITO).

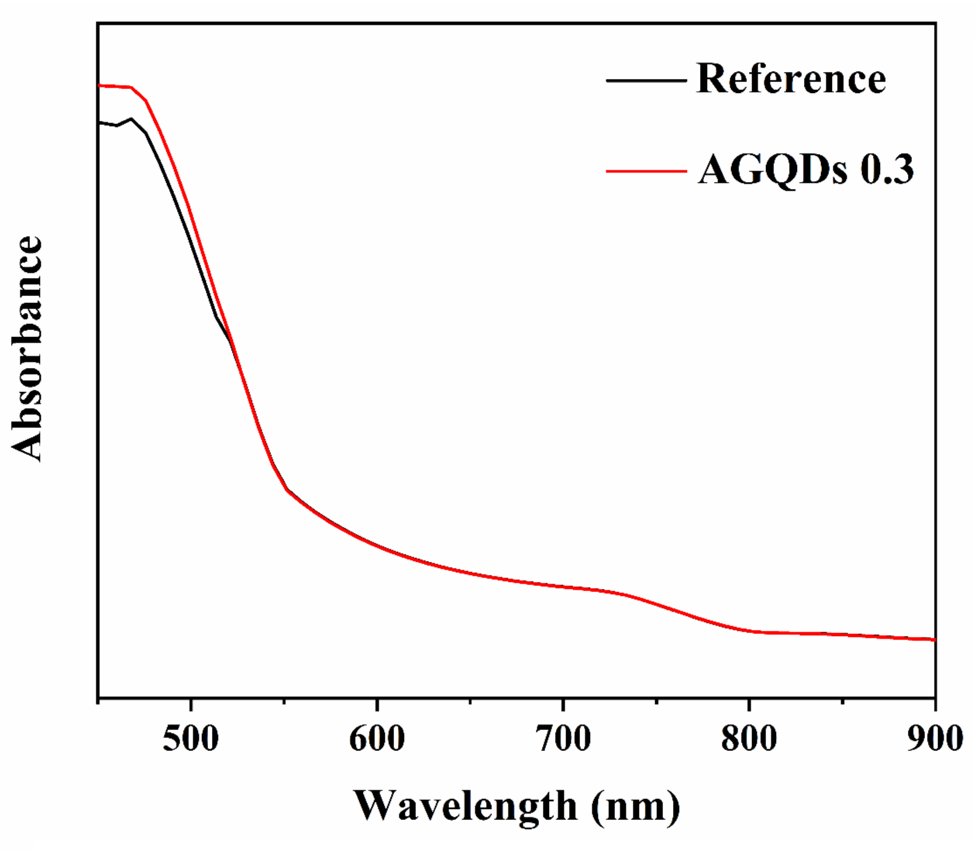

Figure S6. UV-visible spectra of reference and AGQDs 0.3 films.

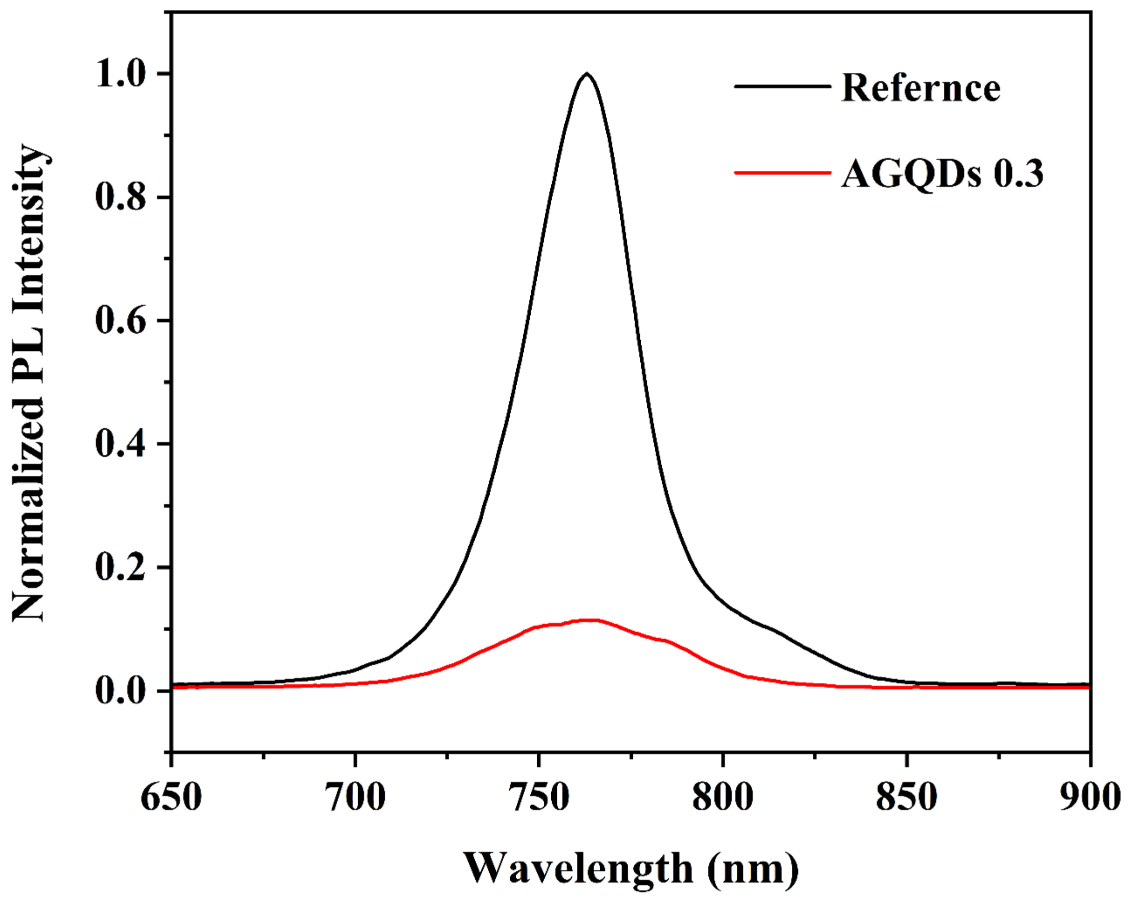

Figure S7. Normalized PL spectra of reference and AGQDs 0.3 perovskite films coated on a glass substrate with spiro-OMeTAD as hole transport layer.

Table S1. The photovoltaic parameters of PSCs based on the corresponding antisolvent: ethyl acetate (Reference) and 0.0, 0.2, 0.3, and 0.4 mL of AGQDs in T:H denoted as AGQDs 0.0, AGQDs 0.2, AGQDs 0.3, and AGQDs 0.4, respectively.

**Devices**		**J_{sc} (mA. cm^{-2})[a]**	**V_{oc} (V)[a]**	**FF[a]**	**PCE (%)[a]**
**Ref**	**Champion**	21.45	1.07	0.70	16.00
	**Average**	21.16 ± 0.75	1.06 ± 0.019	0.67 ± 0.18	15.08 ± 0.38
**AGQDs 0.0**	**Champion**	21.7	1.12	0.7	17.08
	**Average**	21.11±0.50.26	1.09 ± 0.028	0.72 ± 0.027	16.66 ± 0.26
**AGQDs 0.2**	**Champion**	21.92	1.06	0.76	17.69
	**Average**	21.29±0.54	1.09 ± 0.016	0.74 ± 0.013	17.13 ± 0.47
**AGQDs 0.3**	**Champion**	22.07	1.15	0.76	19.10
	**Average**	22.09 ± 0.43	1.11 ± 0.026	0.74 ± 0.012	18.37 ± 0.43
**AGQDs 0.4**	**Champion**	21.93	1.13	0.71	17.55
	**Average**	21.32 ± 0.64	1.10 ± 0.027	0.72 ± 0.011	16.89 ± 0.33

[a] Statistic values were obtained from 11 individual solar cells fabricated in different experimental batches for each sample.
